# Case Report: Severe thrombocytopenia in the context of concomitant tirofiban and ibuprofen use: does ibuprofen matter?

**DOI:** 10.3389/fcvm.2025.1672200

**Published:** 2025-11-18

**Authors:** Juxun Zhu, Chao Chen, Chenlu Zhu, Lili Sun

**Affiliations:** 1 Department of Nursing, The First Affiliated Hospital of Shandong First Medical University & Shandong Provincial Qianfoshan Hospital, Jinan, China; 2 Department of Gerontology, The First Affiliated Hospital of Shandong First Medical University & Shandong Provincial Qianfoshan Hospital, Jinan, China; 3 Department of Neurology, The First Affiliated Hospital of Shandong First Medical University & Shandong Provincial Qianfoshan Hospital, Jinan, China

**Keywords:** thrombocytopenia, tirofiban, drug-induced thrombocytopenia, ibuprofen, anti-inflammatory drugs

## Abstract

Tirofiban is a glycoprotein (GP) IIb/IIIa receptor antagonist that inhibits platelet-to-platelet interactions and thrombosis by preventing fibrinogen from binding to platelets. While it has the potential to cause thrombocytopenia and bleeding, instances of severe thrombocytopenia are rare. The question arises whether the concurrent use of tirofiban with other drugs that pose risks to platelets might increase the likelihood of severe platelet depletion. Herein, we present two cases of profound and sudden thrombocytopenia associated with tirofiban use in the treatment of acute progressive stroke. Both patients received ibuprofen for pain relief concomitantly. Drug-induced thrombocytopenia (DITP), a rare but potentially life-threatening adverse effect, occurred. Ibuprofen is a nonsteroidal anti-inflammatory drugs (NSAIDs) with both aspects regarding platelets: function inhibition, responsible for drug-induced thrombocytopenia. We recommend avoiding concomitant use of ibuprofen in patients receiving tirofiban infusion. Alternative analgesics (e.g., acetaminophen/paracetamol) may be considered when pain management is required. If concurrent administration is unavoidable, intensive platelet count monitoring (e.g., every 6–12 h) is imperative during the first 24 h of therapy.

## Introduction

Drug-induced thrombocytopenia (DITP) is a condition characterized by a decrease in platelet count due to drug exposure, first described in the 19th century (1). Many medications induce thrombocytopenia through immune mechanisms. The incidence of adult DITP is estimated to be approximately 10 per million inhabitants per year (2, 3). Generally, when the platelet count falls below 50 × 10^9^/L, patients are at increased risk of developing spontaneous hemorrhages (3). We present two cases of patients with extremely low platelet counts over a very short period, which we believe to be instances of DITP.

## Methods

### Study patients

We retrospectively reviewed our database to identify patients who had been treated with tirofiban between January 2013 and December 2023. All patients or their legal guardians knew the risks and benefts of tirofban and gave informed consent. The study protocol was approved by the ethics committee of the First Afliated Hospital of Shandong First Medical University and complied with the Declaration of Helsinki. All research was performed under the relevant guidelines and regulations. Given its retrospective nature, the study does not require registration.

### Tirofiban

In our center, the application of tirofiban (Shandong New Era Pharmaceutical Co., Ltd., Shandong, China) was as follows: Tirofiban was administered intravenously at a dose of 0.4 µg × kg^−1^ × min^−1^ for 30 min followed by 0.1 µg × kg^−1^ × min^−1^ for up to 10–24 h.

### Ibuprofen

The first patient in this study used ibuprofen tablets produced by Shandong Xinhua Pharmaceutical Co., Ltd. Each tablet contains 0.2 g of the main ingredient ibuprofen, with one tablet per dose for adults. If there is persistent pain or fever, the medication can be repeated once every 4–6 h, and no more than 4 times within 24 h. The second patient used ibuprofen sustained-release capsules produced by China US Tianjin Square Pharmaceutical Co., Ltd. Each capsule contains 0.3 g of the main ingredient ibuprofen, one capsule for adults, twice a day (once in the morning and once in the evening.

## Exclude EDTA dependent pseudothrombocytopenia

Ethylenediaminetetraacetic acid dipotassium (EDTA-K2) anticoagulant blood routine examination was used. Retested with sodium citrate and respective blood smears were examined by optic microscope to rule out tirofiban-induced false thrombocytopenia. (Automated hematology analyzer, Sysmex XN-9000).

## Case presentation

### Patient 1

**Figure 1:**
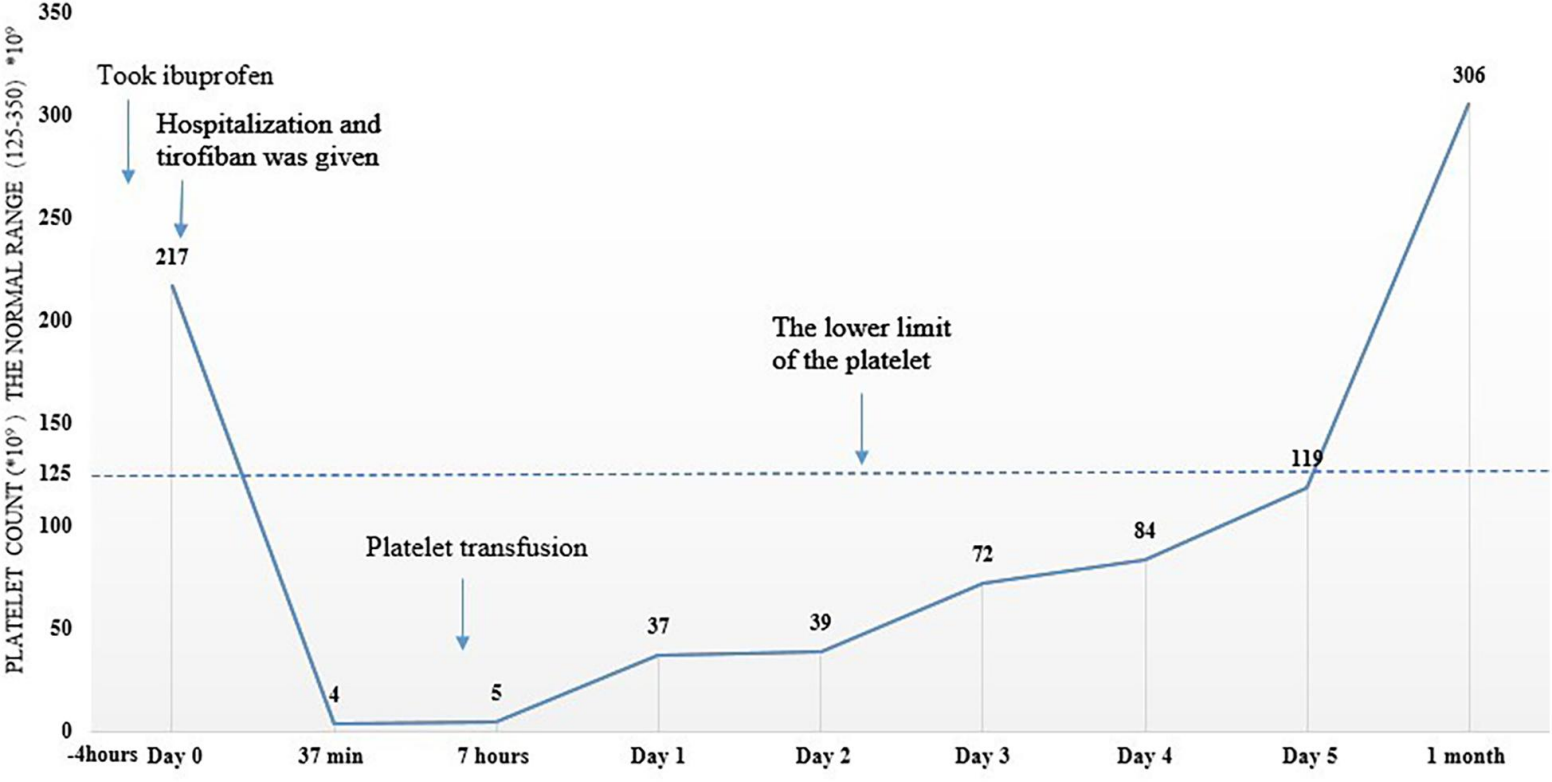
Changes in platelet count over time of the first patient. The patient took ibuprofen before admission. Platelets are normal on admission. However, platelet count drop dramatically in a very short period after intravenous infusion of tirofiban.

A 52-year-old male patient, with no significant past medical history, was admitted with symptoms of dizziness, nausea, headache, and vomiting persisting for more than 20 h. Cerebral magnetic resonance imaging (MRI) detected acute/subacute cerebral infarction in the left cerebellar hemisphere and cerebellar vermis, multiple ischemic infarcts in the brain, and severe stenosis or occlusion of the right middle cerebral artery. Prior to hospital admission, the patient took ibuprofen to relieve his headache. His platelet count was normal (217 × 10^9^/L) before dosing. He was treated with 0.1 g of aspirin and 300 mg of clopidogrel daily. Tirofiban was administered intravenously at a dose of 0.4 µg/kg/min for 30 min, followed by 0.1 µg/kg/min. After 37 min of tirofiban infusion, the patient developed epigastric discomfort, chest tightness, lip cyanosis, and chills. The patient's axillary temperature was 38.8 °C, arterial oxygen saturation at the fingertip was approximately 90%, and blood pressure was 146/73 mmHg. Acute allergic reaction was prime considerations. Tirofiban was discontinued and Dexamethasone (5 mg) was administered intravenously. Urgent blood routine examination revealed a rapid drop in platelet count to 4 × 10^9^/L. Aspirin and clopidogrel were discontinued immediately, and methylprednisolone sodium succinate (40 mg) was administered via intravenous drip. One unit of platelets (2.5 × 10^11^, 250 mL, Leukocyte-depleted irradiated apheresis platelets) was transfused, and pantoprazole (40 mg) was given twice a day. After 3 h, the platelet count was 5 × 10^9^/L, and it gradually returned to normal over the next 5 days. Aspirin and clopidogrel were resumed when the platelet count exceeded 84 × 10^9^/L. Throughout this period, the patient did not exhibit any signs of bleeding and was safely discharged. He was followed up regularly in the outpatient clinic for one year, during which the platelet count remained stable. Changes in platelet count are shown in Figure 1.

### Patient 2

A 44-year-old female patient with a three-year history of hypertension and hyperlipidemia was admitted to our Department of Neurology for follow-up after cerebral artery stent implantation. Six months prior, the patient had been admitted with episodic right limb weakness and underwent stenting for severe stenosis of the C7 segment of the right internal carotid artery. Tirofiban was administered intravenously at a dose of 0.15 µg/kg/min immediately after femoral artery puncture and continued for 14 h postoperatively. During this period, no reduction in platelet count was observed. After intracranial stent angioplasty, aspirin 0.1 g once daily and ticagrelor 90 mg twice daily were administered regularly. During this admission, the patient experienced frequent seizures of left limb numbness and weakness, which were relieved after a few minutes. Considering the patient's multiple cerebral artery stenosis and occlusion, poor vascular condition, frequent occurrence of transient ischemic attacks (TIA), and high risk of symptom aggravation, tirofiban was initially administered at 0.1 µg/kg/min intravenously for 30 min, followed by 0.05 µg/kg/min. Following this, the patient did not experience left limb numbness and weakness, and tirofiban was discontinued 17 h later. The platelet count was rechecked and found to be 219 × 10^9^/L, within the normal range. For further diagnosis, digital subtraction angiography (DSA) was performed, revealing severe in-stent restenosis of the internal carotid artery, occlusion of the initial segment of the left middle cerebral artery, occlusion of the A2 segment of the right anterior cerebral artery, and severe stenosis of the A1 segment of the left anterior cerebral artery.

**Figure 2:**
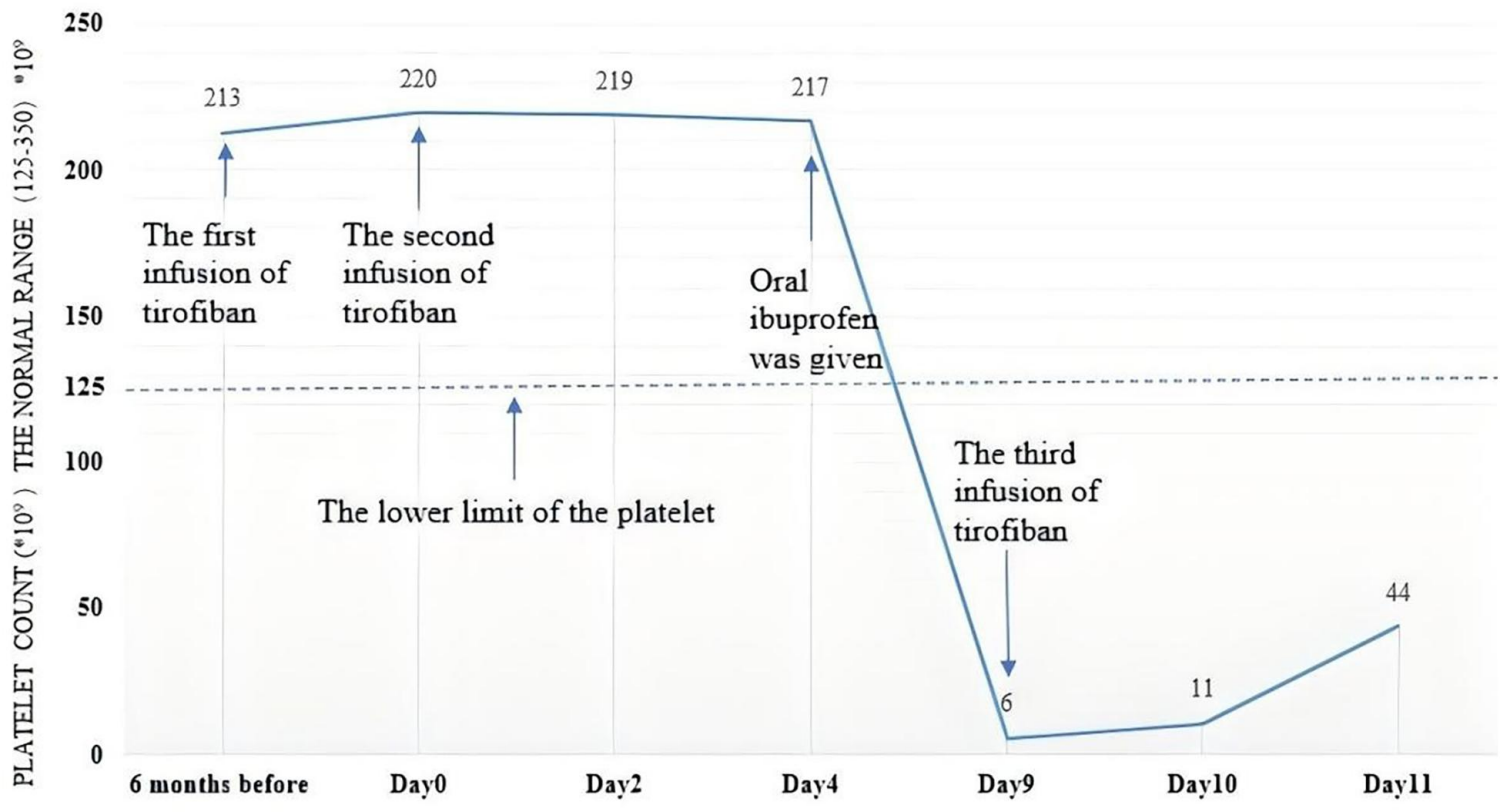
Changes in platelet count over time of the second patient. The patient had normal platelet count even after infusion of tirofiban twice. However, the platelet count decreases fatally after being used in combination with ibuprofen.

Tenderness was complained in the right groin puncture site after DSA. Ultrasound revealed a mixed echo mass in the right intrapelvic parailiac region, suggesting a hematoma. Blood routine reexamination did not indicate progressive bleeding. After consultation with a vascular surgeon, the patient was prescribed oral ibuprofen 0.3 g twice daily for analgesia. Five days post-DSA, the patient developed paroxysmal numbness on the right side of the face and limb. Brain computed tomography revealed no intracerebral hemorrhage, and tirofiban was re-administered intravenously at 0.05 µg/kg/min. Eighteen hours later, routine testing showed the patient's platelet count had dropped to 6 × 10^9^/L. The patient's axillary temperature was 37.8 °C, arterial oxygen saturation was 99%, and blood pressure was 136/72 mmHg. Tirofiban, ibuprofen, and antiplatelet drugs were discontinued, and recombinant human thrombopoietin (rhTPO, Shenyang Sansheng Pharmaceutical, China; brand name: Tebiao) was administered once at a dose of 300 U × kg^−1^ subcutaneously. The next day, the patient's platelet count rose to 44 × 10^9^/L, but she developed a massive cerebral infarction. Two days later, the patient died. Changes in platelet count are shown in Figure 2.

Recombinant human thrombopoietin is administered by subcutaneous injection once, at a dose of 300 U/kg.

## Discussion

Thrombocytopenia may have negative effects on clinical outcomes, yet clinicians have difficulty identifying high-risk patients with thrombocytopenia. Many drugs can cause thrombocytopenia either by non-immune or immune mechanisms. DITP is a challenging clinical problem that is under-recognized, difficult to diagnose and associated with severe bleeding complications (4). Therefore, it may be crucial to identify patients at high risk of thrombocytopenia for early and timely intervention. The medical records revealed that neither patient had previously experienced thrombocytopenia, and their initial blood tests upon admission showed normal platelet levels. What was the true reason for the rapid decrease in platelet counts? Excluding primary thrombocytopenia, thrombocytopenia in the context of concomitant tirofiban and ibuprofen use suggests a potential interaction between them.

When the first case emerged, we focused significant attention on it. After analyzing the patient's current medications and reviewing the literature, we identified tirofiban, a platelet glycoprotein (GP) IIb/IIIa receptor antagonist, as a potential cause. Tirofiban competitively inhibits platelets GpIIb/IIIa receptor and removes fibrinogen, resulting in disintegration of a hyperacute thrombus (5).

Clinical studies have demonstrated that tirofiban can enhance cerebral artery recanalization and tissue reperfusion, thereby reducing infarct volume and improving neurological prognosis (6). It is beneficial for the prognosis of AIS-END patients who missed the intravenous thrombolytic time window (7). Additionally, multiple studies have indicated that tirofiban can significantly improve patient outcomes in the interventional treatment of cerebrovascular diseases (8–10). Consequently, tirofiban has been increasingly utilized in recent years for the treatment of cardiovascular and cerebrovascular diseases. Although cases of thrombocytopenia induced by tirofiban have been reported, occurrences of extremely low platelet counts are rare (11). In clinical trials, the incidence of severe thrombocytopenia was reported to be between 0.1% and 0.5% (12). Severe thrombocytopenia (<20 × 10^9^/L) is relatively uncommon. In this case, the patient's minimum platelet count was 4 × 10^9^/L. Upon discontinuation of tirofiban, the patient's platelet levels gradually normalized. However, the specific mechanism behind tirofiban-induced thrombocytopenia remains unclear. Possible mechanisms include tirofiban binding to platelet GP IIb/IIIa receptors, inducing conformational changes in these receptors, forming new antigenic determinants, increasing affinity for existing drug-dependent antibodies, and promoting their binding to new antigenic determinants, thereby accelerating platelet destruction and clearance (13).

We encountered a second patient who had received intravenous tirofiban for 14 h following cerebrovascular stent implantation six months prior, without a decrease in platelet count. During the current admission, tirofiban was administered intravenously for 17 h, and the patient's platelet count remained normal. Thrombocytopenia occurred in the context of using tirofiban and ibuprofen (0.3 g twice daily ibuprofen for analgesia and then tirofiban for the worsening of cerebral infarction).

Does this patient also have thrombocytopenia due to tirofiban? Why did the patient not experience a decrease in platelet count on two previous doses of the drug? Upon reviewing the medical records, we found that the only difference was that the patient was also administered ibuprofen orally for pain during the intravenous injection of tirofiban. We revisited Case 1 and made a significant discovery: Case 1 also took ibuprofen for a headache four hours before admission. Is ibuprofen the sole factor causing thrombocytopenia in patients?.

After reviewing the literature, we found that thrombocytopenia is a rare side effect of some nonsteroidal anti-inflammatory drugs (NSAIDs), including diclofenac, naproxen, and ibuprofen (14, 15). Ibuprofen mainly exerts its anti-inflammatory, analgesic, and antipyretic effects by inhibiting cyclooxygenase (COX) (16). There are two isoenzymes of cyclooxygenase: COX-1 and COX-2. COX-1 is expressed in platelets and promotes platelet aggregation. The primary mechanism by which NSAIDs interfere with platelet function is through the reversible inhibition of COX-1 enzyme activity within platelets. This leads to a reduction in the synthesis of prostacyclin (PGI2) and thromboxane A2 (TXA2), both of which normally play important roles in vascular dilation and platelet aggregation (17).

A cohort study of rare serious adverse events associated with steroidal anti-inflammatory drugs (steroids) at Boston University Medical Center reported multiple cases of severe thrombocytopenia caused by steroids (18). Meyer et al. reported a case of a 71-year-old woman with no history of hematologic disorders who was admitted to the hospital with extensive hemorrhagic purpura, petechiae, and epistaxis following immune thrombocytopenia caused by ibuprofen (19). Medford et al. reported a case of a 71-year-old African American male who developed thrombocytopenia, autoimmune hemolytic anemia, acute liver failure, and interstitial nephritis associated with prolonged ibuprofen use (20). Ibuprofen can cause immune thrombocytopenia through one of its metabolites. A systematic evaluation of laboratory testing for DITP in Canada reported that 16 drugs met the criteria for a definite laboratory diagnosis of DITP, including ibuprofen (21). Ibuprofen is widely used to manage minor aches and pains, reduce fever, and relieve symptoms of dysmenorrhea (22). Since the outbreak of COVID-19 in 2019, it has been recommended for controlling symptoms of the virus (23).

In our study, both patients had taken ibuprofen several times before without experiencing thrombocytopenia. Therefore, we believe that ibuprofen should not be the only factor causing extreme thrombocytopenia. The similarity in the cases leads us to suspect that the simultaneous use of ibuprofen and tirofiban may increase the risk of extreme platelet count reductions. However, we do not understand the specific mechanism; this clinical observation is difficult to determine whether it is merely coincidental. Both laboratory studies in healthy volunteers and clinical studies have suggested adverse interactions between antiplatelet drugs and other commonly used medications. Both the aspirin- and the NSAID-binding sites lie within a narrow hydrophobic channel within the core of the enzyme. The potential for a competitive interaction between aspirin and NSAIDs afforded by these structural relations is supported by evidence from previousstudies (24, 25). Ibuprofen inhibits platelet aggregation by transiently blocking COX-1–mediated thromboxane A₂ synthesis, which may increase bleeding risk. Moreover, ibuprofen can interfere with the irreversible COX-1 inhibition produced by aspirin, as demonstrated in the pivotal study by Catella-Lawson et al. (26).

The ibuprofen/aspirin interaction is thought to be caused by ibuprofen blocking the access of aspirin to platelet cyclo-oxygenase (27). Althouth, there is relatively little literature on drug-drug interactions of tirofiban/Ibuprofen leading to DITP. The co-administration of ibuprofen with tirofiban may have had a similar functional additive effect, contributing to the severity of thrombocytopenia and bleeding risk. With numerous therapies shown to be effective in common conditions, this has resulted, and will continue to result, in poly-therapy being the norm for patients. This means that the opportunity for drug interactions will inexorably increase. We need to be vigilant in detecting these interactions.

Once DITP is recognized, the offending drug should be discontinued, and other antithrombotic agents should be adjusted according to the presence or absence of bleeding complications. If the platelet count is below 10 × 10^9^/L and the risk of bleeding is high, platelet transfusion and thrombopoietin therapy may be considered (28, 29). Infusing platelets and using thrombopoietin may lead to stent thrombosis, acute myocardial infarction, and cerebral infarction (30), as seen in our second patient who developed a life-threatening massive cerebral infarction after thrombopoietin therapy. Therefore, the benefits and risks must be carefully weighed when considering such treatments. For patients whose platelet count remains consistently low without recovery after platelet transfusion, immunoglobulin therapy may be considered (31). When using drugs that pose a risk of DITP, it is recommended to test the platelet count within 2–6 h after starting the drug and then daily, to detect most cases of thrombocytopenia promptly. It is also advised to avoid taking multiple drugs that carry the risk of thrombocytopenia simultaneously to prevent exacerbating the condition.

The common feature in these two cases is that a rapid and extreme drop in platelet count after concomitant use of tirofiban and ibuprofen. This suggests the need for caution when using tirofiban alongside ibuprofen. We recommend monitoring platelet counts and observing for bleeding tendencies in patients concomitant use of tirofiban and ibuprofen. Alternative analgesics (e.g., acetaminophen/paracetamol) should be considered when pain management is required. If concurrent administration is unavoidable, intensive platelet count monitoring (e.g., every 6–12 h) is imperative during the first 24 h of therapy. If both drugs must be used, the patient's platelet count should be monitored closely and frequently to detect any extreme decreases and mitigate the risk of bleeding.

## Conclusion

Drug-induced thrombocytopenia (DITP) is a rare but potentially dangerous adverse effect. DITP can be life-threatening. Two cases of severe thrombocytopenia in the context of concomitant tirofiban and ibuprofen use suggest a potential interaction between them. We recommend monitoring platelet counts and observing for bleeding tendencies in patients concomitant use of tirofiban and ibuprofen.

## Data Availability

The original contributions presented in the study are included in the article/Supplementary Material, further inquiries can be directed to the corresponding author.
